# Direct and Indirect Effects of Environmental Limitations on White Spruce Xylem Anatomy at Treeline

**DOI:** 10.3389/fpls.2021.748055

**Published:** 2021-10-25

**Authors:** Timo Pampuch, Alba Anadon-Rosell, Mario Trouillier, Jelena Lange, Martin Wilmking

**Affiliations:** ^1^Institute of Botany and Landscape Ecology, University Greifswald, Greifswald, Germany; ^2^CREAF – Centre for Research on Ecology and Forestry Applications, Barcelona, Spain; ^3^Department of Physical Geography and Geoecology, Charles University in Prague, Prague, Czechia

**Keywords:** boreal forest, conduit reinforcement, drought limitation, hydraulic stability, *Picea glauca*, temperature limitation, tree allometry, tree height

## Abstract

Treeline ecosystems are of great scientific interest to study the effects of limiting environmental conditions on tree growth. However, tree growth is multidimensional, with complex interactions between height and radial growth. In this study, we aimed to disentangle effects of height and climate on xylem anatomy of white spruce [*Picea glauca* (Moench) Voss] at three treeline sites in Alaska; i.e., one warm and drought-limited, and two cold, temperature-limited. To analyze general growth differences between trees from different sites, we used data on annual ring width, diameter at breast height (DBH), and tree height. A representative subset of the samples was used to investigate xylem anatomical traits. We then used linear mixed-effects models to estimate the effects of height and climatic variables on our study traits. Our study showed that xylem anatomical traits in white spruce can be directly and indirectly controlled by environmental conditions: hydraulic-related traits seem to be mainly influenced by tree height, especially in the earlywood. Thus, they are indirectly driven by environmental conditions, through the environment’s effects on tree height. Traits related to mechanical support show a direct response to environmental conditions, mainly temperature, especially in the latewood. These results highlight the importance of assessing tree growth in a multidimensional way by considering both direct and indirect effects of environmental forcing to better understand the complexity of tree growth responses to the environment.

## Introduction

Boreal forests play a crucial role for the global carbon cycle, are an important source for timber and non-timber products and provide several ecosystem services (Arneth et al., 2010; Gauthier et al., 2015; Tagesson et al., 2020). Yet they are particularly challenged by global warming. The effects of global warming are assumed to be more severe in areas of high latitude (Soja et al., 2007; Charney et al., 2016; IPCC, 2021), such as parts of Canada and Alaska, and will certainly have a lasting impact on boreal forest ecosystems. Especially trees that are growing under marginal conditions, i.e., at treelines, will be influenced by climate change. Treelines are characterized as the edge of the habitat at which trees are able to grow. This edge is caused by environmental limitations like low temperatures or drought (Brockmann-Jerosch, 1919; Däniker, 1932). With climate change, the conditions at treelines are expected to shift, with drastic consequences for tree growth, spatial distribution and dynamics of treeline populations (Grace et al., 2002; Wieser, 2020). In order to evaluate how trees at treelines respond to these rapid shifts, we need to understand the impacts of changing environmental conditions on tree growth.

White spruce is one of the most important and widespread tree species of the North American boreal forest (Little and Viereck, 1971). It grows under a variety of environmental conditions, making it a model organism for investigating the influence of climate on tree growth (Lloyd and Fastie, 2002; Wilmking and Juday, 2005; Sherriff et al., 2017). Most previous studies on white spruce have focused on the growth performance and total annual growth using tree ring-based analyses. However, to understand the complexity of growth and how environmental conditions influence trees in multiple ways, more holistic approaches at intra-annual resolution are needed, e.g., by including investigations on phenology, xylogenesis or xylem anatomy. While collecting data on phenology and xylogenesis is labor intensive and requires regular monitoring, xylem anatomical analyses can be performed retrospectively using typical increment cores. Xylem is multifunctional as it is responsible for conducting water from the roots, through the stem to the leaves and to provide the structural support of a tree (Domec and Gartner, 2002; Hacke et al., 2015). The benefit of studying the structure of this multifunctional tissue is that it can provide deeper insights into inter- and intra-annual variation of growth and how different parts of a tree ring are influenced by environmental conditions and by biotic factors at different times (Fonti et al., 2010; von Arx et al., 2016). On the one hand, it is well known that xylem anatomical traits that are linked to water transport, like lumen area, are influenced by tree height, especially in earlywood (Callaham, 1962; Anfodillo et al., 2006; Carrer et al., 2015). With up to 90% of the total water flow, water transport is mainly occurring through the earlywood tracheids (Domec and Gartner, 2002), making the structure of the earlywood tracheids potentially sensitive to changes in the water availability. Tracheids with smaller lumen are known to occur as an adjustment toward dry conditions to avoid drought induced cavitation (Cochard, 2006; Eldhuset et al., 2013) and thus drought-induced damage to the tree. Yet to secure a safe water transport in the earlywood, also the so-called conduit widening is an important driver that influences the tracheid structure. Conduit widening means that with increasing vertical path length (i.e., tree height) the lumen area widens from the apex to the base to maintain a constant hydraulic resistance along the path (Sanio, 1872; Mencuccini et al., 2007; Anfodillo et al., 2013; Kašpar et al., 2019). On the other hand, traits related to structural integrity (i.e., the ability of a structure to support a certain weight and/or force without being damaged) like xylem cell wall thickness (CWT) are largely driven by climatic conditions, especially in latewood (Antonova and Stasova, 1993; Yasue et al., 2000; Fonti et al., 2013; Cuny and Rathgeber, 2016). In the latewood structure of conifers, water transport is less important and cell wall deposition take place to a greater extent. Cell wall deposition and thus the resulting thickness of the cell wall is a temperature sensitive process. With higher temperatures, the processes can occur faster and thicker cell walls might be build and vice versa, making the latewood potentially more sensible toward climatic changes (Yasue et al., 2000; Fonti et al., 2013; Björklund et al., 2020). However, different xylem anatomical traits are also linked to each other and function in multiple dimensions. For example, although lumen area is mainly influenced by tree height and CWT is mainly influenced by climatic conditions, CWT is also linked to lumen area, due to physiological limitations. I.e., if a certain amount of resources is available for cell wall deposition, the maximum functional cell-size (i.e., functional in way that the cell is capable to efficiently transport water and provide structural support) is limited by this amount. Building smaller cells using the exact same amount of resources would result in cells with thicker walls, due to a surplus of available material (Cuny et al., 2014). This relation inevitably influences traits that are derived both from cell wall and lumen measurements, such as wood density and the conduit reinforcement index (Hacke and Sperry, 2001; Hacke et al., 2001; Pittermann et al., 2006). This complexity makes the analysis of these traits challenging and requires careful interpretation. A comprehensive study approach that combines xylem anatomical investigations with modeling helps to disentangle and better understand the underlying patterns.

Here, we investigate the multifunctionality (i.e., water transport and structural integrity) and multidimensionality of xylem anatomy in white spruce by studying the effects of tree height (i.e., vertical dimension), precipitation, and temperature on four xylem anatomical traits (lumen area, conduit reinforcement index, anatomical wood density, and CWT) separated into earlywood and latewood (i.e., temporal dimension) of 18 individuals from three treeline sites (two temperature-limited and one drought-limited) in Alaska. We expected to find that (1) earlywood traits related to water transport would be strongly influenced by tree height due to the importance of earlywood for water transport, while (2) latewood traits related to structural integrity would be mainly influenced by climatic conditions, since climate-sensitive processes like cell wall deposition have a larger effect during latewood formation. We additionally assumed that trees from the drought-limited treeline would have (3) smaller lumen areas compared to trees from the temperature-limited sites as a strategy to lower the risk of cavitation (Cochard, 2006; Eldhuset et al., 2013) and (4) thicker cell walls due to the higher temperatures during the vegetation period, as well as longer vegetation periods at the drought-limited treeline, which would lead to an increased level of cell wall deposition (Fonti et al., 2013; Björklund et al., 2020).

## Materials and Methods

### Study Species and Study Sites

**FIGURE 1:**
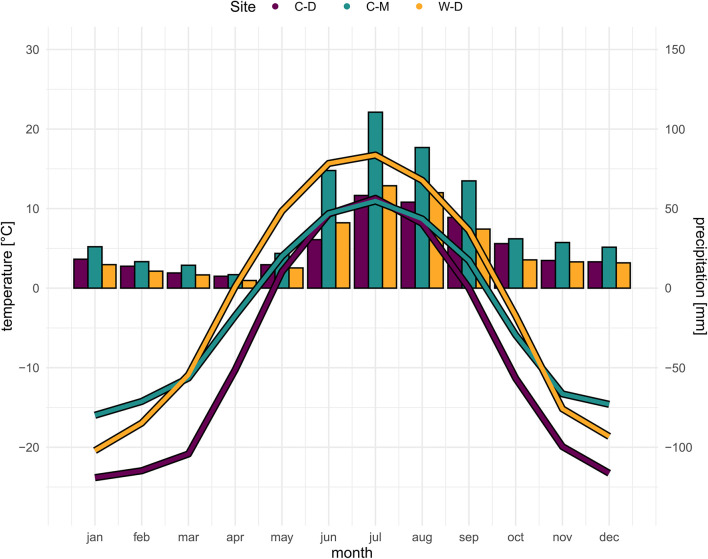
Site-specific climatic conditions (SNAP, 2019). Bars showing precipitation sum per month, curve showing average temperature per month for the period 1970–2016. Purple = Brooks Range (C-D), Green = Denali National Park (C-M), Orange = Bluff (W-D).

White spruce [*Picea glauca* (Moench) Voss] is a widely distributed tree species in the boreal forests of North America. It is of large economic importance (Attree et al., 1991) and its distributional range covers most of the boreal area in Canada and Alaska, and parts of the northernmost US mainland (Little and Viereck, 1971; Supplementary Figure 1). White spruce is the dominant tree species at the elevational and latitudinal treeline in the north-western parts of its distributional range (Nienstaedt and Zasada, 1990; Abrahamson, 2015). We sampled white spruce trees at three study sites (Supplementary Figure 1), one treeline site where tree growth is mainly limited by drought and two treeline sites where tree growth is mainly limited by low temperatures. The presumably drought-limited treeline is located in Interior Alaska at a steep (12–34°) bluff of the Tanana River near Fairbanks—Warm—Dry Treeline (W-D; 64°42′ N, 148°18′ W). W-D is characterized by a very low precipitation but relatively high temperatures during the vegetation period (Figure 1), causing a potential water limitation (Barber et al., 2000; Juday et al., 2003; Lloyd et al., 2013). The first temperature-limited study site is a latitudinal treeline located on a slope of Nutirwik Creek valley in the central Brooks Range—Cold—Dry Treeline (C-D; 67°56′N, 149°44′W). At C-D, tree growth is mainly limited by low temperatures (Wilmking et al., 2017), yet the mean annual precipitation is comparably low as well, which could lead to occasional drought stress (Figure 1; Trouillier et al., 2019). The second temperature-limited site is in Denali National Park—Cold—Moist Treeline (C-M, 63°43′N, 149°00′W). At C-M, growth is mainly limited by low temperatures as it is a typical elevational treeline (Körner, 2007), while the precipitation is relatively high in comparison to the other study sites (Figure 1). All three study sites are located on south-facing slopes. For more details on the characteristics of the study sites see also Trouillier et al. (2018) and Zacharias et al. (2021).

### Climate Data

Modeled historical monthly climate data (i.e., mean temperature and total precipitation sums) for the period of 1901–2015 were obtained from the Scenarios Network for Alaska and Arctic Planning at 1 × 1 km spatial resolution (SNAP, 2019; Figure 1). The climate data is based on a General Circulation Model (GCM) data set that was downscaled to 2 × 2 km gridded climate data using a Weather Research and Forecasting Model (WRF; Bieniek et al., 2016). The downscaled 2 × 2 km data was then resampled to a 1 × 1 km spatial resolution using bilinear interpolation (SNAP, 2019). To reduce the number of parameters in the models we averaged the mean temperature and total precipitation for each of the four seasons of the year. Tree growth at our study sites typically starts in May/June and presumably ceases toward the end of August (Ueyama et al., 2013), thus the seasons used in the analysis were defined as follows: previous fall (i.e., previous year September to previous year November), previous winter (i.e., previous year December to current year February), spring (March to May) and summer (June to August).

### Sampling and Data Acquisition

Increment cores were originally collected in 2012 (Eusemann et al., 2016), 2015 and 2016 (Wilmking et al., 2017) from 1254 trees (W-D: 309 trees, C-D: 474 trees, C-M: 471 trees). We documented the diameter at breast height (DBH) and the total height of each individual once at the moment it was sampled. These samples were used for the general diameter-height analysis. All cores were taken with a 4.3 mm Haglöf increment borer (Haglöf, Sweden). Cores were air dried and glued onto wooden sample holders. The surfaces were either polished with progressively finer sandpaper (up to 800 grit) or cut with a core-microtome (Gärtner and Nievergelt, 2010), before they were digitized with a flatbed scanner (Epson Perfection V700 Photo; Seiko Epson Corporation, Japan) with 3,200 dpi. Tree-ring widths (TRW) were subsequently measured using CooRecorder (version 9.3.1; Cybis Elektronik and Data AB, Sweden) and all radii were cross-dated using CDendro (version 9.3.1; Cybis Elektronik and Data AB, Sweden).

To explore how tree growth differs between sites, we explored the relationship between tree’s age, DBH and height: First, we fitted a modified Weibull function for each site to describe the relationship between DBH and tree height (Huang et al., 1992):


Equation1        ht=hmax*e(a*DBHtb),

where h_t_ is the current tree height, h_max_ is the maximum height (asymptote) that a tree approaches, while the parameters a and b modulate how this asymptote is approached. The parameters were estimated in R v4.0.2 (R Core Team, 2020) with the nls2 R-package (Grothendieck, 2013). Next, we used the rcs function from the dplR package (Bunn, 2008) to estimate the relationship between ring width and tree age. Because the cumulative ring width is (half) the stem diameter, this function can also describe the stem diameter-age relationship. Thus, combined these functions describe the relationship between age, diameter and height for each site, which is the tree’s ontological development over time. We used this relationship for the visualization of site differences and to guide the interpretation of the results.

For xylem anatomical analysis, six samples were selected each from the samples collected in 2012 at W-D and in 2015 at C-D. Six additional samples were collected in 2018 at C-M. All trees used for xylem anatomical analysis were selected from the available samples based on their height, being approximately similar (Supplementary Table 1). The samples were handled the same as the aforementioned samples of the entire set. Afterward we cut 12 μm-thin cross-sections from one radius of each tree using a rotary microtome (Leica RM 2245; Leica Camera AG, Germany). The cross-sections were stained with 1:1 safranin and astrablue solution, rinsed with ethanol solutions of increasing concentration (50, 70, 96%), mounted on microscope slides with Euparal and dried at 60°C for 48 h (Gärtner and Schweingruber, 2013). The slides were digitized with a Leica DFC450C camera installed on a Leica DM2500 microscope with a 10 × magnification objective (Leica 506505). We used the digitized images to quantify TRW and the xylem anatomical traits lumen area (LA), lumen diameter (LD), cell wall area (CWA), radial, tangential, mean CWT and conduit reinforcement index (TB^2^) with the image analysis tool ROXAS v3.0.326 (von Arx and Carrer, 2014; Prendin et al., 2017).

TB^2^ was calculated in ROXAS based on Hacke et al. (2001):


$$\mathrm{Equation2}\;{\mathrm{TB}}^{2}={\left(\frac{T}{B}\right)}^{2},$$

where *T* is double CWT and *B* the length of the same cell wall. For each cell, the smaller of radial and tangential CWT measurements was selected (Hacke et al., 2001; von Arx and Carrer, 2014; Prendin et al., 2017). We approximated wood density (DEN) as the proportion of the estimated CWA to the total cell area (only including tracheid cells; i.e., the sum of CWA and LA; Eq. 3) according to Björklund et al. (2017):


$$\mathrm{Equation3}\;\mathrm{DEN}=\frac{\mathrm{CWA}}{\mathrm{CWA}+\mathrm{LA}}$$

From all xylem anatomical traits, we selected two traits related to water transport (LA and TB^2^) and two traits related to structural integrity (DEN and CWT) for further analysis. LA and CWT represent the two main functions of xylem tissue in conifers and their use has been reported in several previous studies (Prendin et al., 2017; Cuny et al., 2019; Puchi et al., 2020). TB^2^ and DEN were chosen to represent those functions as well, while also including the link between LA and CWT to incorporate the multifunctionality of xylem tissue (Hacke et al., 2001; Myburg et al., 2013; von Arx and Carrer, 2014; Björklund et al., 2017). We used the measurements of lumen diameter and CWT to distinguish between early and latewood using Mork’s index (Denne, 1989) and calculated annual means separately for earlywood and latewood for LA, TB^2^, DEN and CWT using R v4.0.2 (R Core Team, 2020).

**FIGURE 2:**
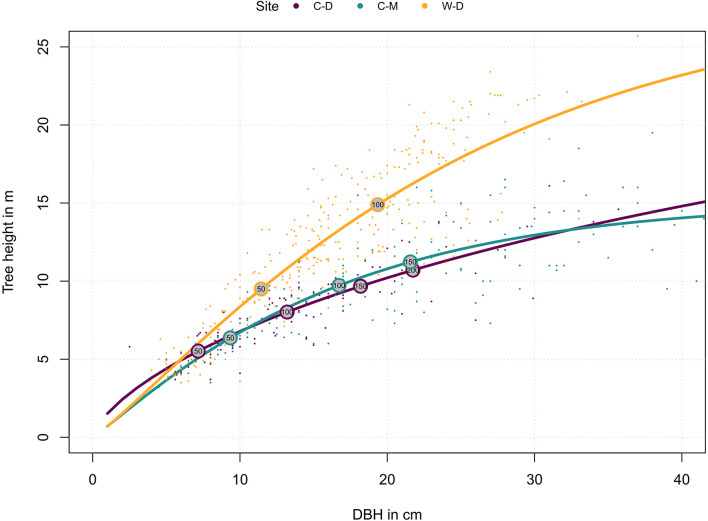
Relationship of site-specific diameter at breast height (DBH) and tree height. Dots represent individual trees. Lines represent the site-specific means as described by the functions in the methods (Eqs. 1 and 2). Numbers in the circles indicate tree age. Purple = Brooks Range (C-D), Green = Denali National Park (C-M), Orange = Bluff (W-D).

For the analysis on the selected xylem anatomical traits, we calculated individual tree height for each year (h_*i*_) for each of the 18 trees that were used for anatomical investigations. In contrast to the calculations for the entire dataset, h_*i*_ for the xylem anatomy analysis was estimated based on a linear function of DBH. We used this linear approach to achieve a potentially more precise calculation of the approximate height for each tree and each year (and thus potentially more precise models), as we accounted for missing rings and the distance to pith from the first measured ring. The method was only valid because tree height of the trees sampled for xylem anatomical analysis was relatively low at the time of sampling and still in the rising leg of the asymptote characteristic for each stand (Supplementary Table 1 and Figure 2). Annual DBH was calculated by subtracting double ring width cumulatively for each year until the first measured year. Missing rings were estimated by averaging the ring width 10 years before and 10 years after the missing ring. For height estimations we then used the coring height as the initial height of the tree with a hypothetical DBH of zero (i.e., the intercept) and tree height measured in the year of sampling as height of the youngest measured ring. We then interpolated h_*i*_ for each year based on a linear function of DBH (Eq. 4).


$$\mathrm{Equation4}\;h_{\mathrm{it}}=h_{\mathrm{i0}}+a_{i}*{\mathrm{DBH}}_{\mathrm{it}},$$

where *h*_*it*_ is the height of tree *i* at time *t*, *h*_*i0*_ is the interecept (i.e., the initial height = coring height), *a*_*i*_ the slope for tree *i* and *DBH*_*it*_ the previously estimated *DBH* of tree *i* at time *t* (Supplementary Figure 2).

### Statistics

**FIGURE 3:**
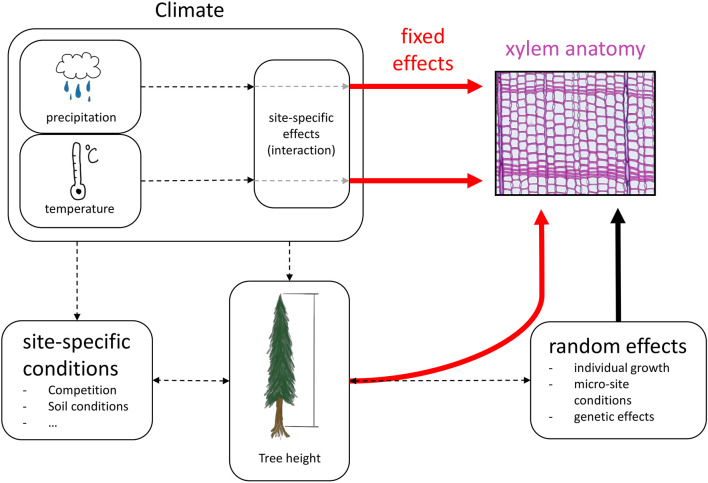
Conceptual design of the linear mixed-effects model. Red arrows indicate fixed effects, the black arrow indicates random effects and black dashed arrows show interactions and effects that are not quantified in the models.

For all statistical analyses we used R v4.0.2 (R Core Team, 2020). We calculated linear mixed-effects models to estimate the effects of tree height and site-specific climatic conditions on the four selected xylem anatomical traits, separated into earlywood and latewood. Xylem anatomical traits were used as response variable (i.e., LA, TB^2^, CWT, DEN). h_*i*_, squared h_*i*_ (h_*i*_^2^), site and the climate variables mean temperature and total precipitation of previous fall, previous winter, spring, and summer were set as fixed effects including interactions between site and each climate variable to account for potential differences in site-specific responses to climatic conditions (Figure 3). h_*i*_^2^ was included in the fixed effects to allow for potential non-linear effects of h_*i*_ on xylem anatomical traits (e.g., LA; Anfodillo et al., 2006). TB^2^ and CWT were log-transformed before the analysis to better meet the assumptions of normality and homoscedasticity of residuals. All investigated numeric response and explanatory variables were standardized to a mean of 0 with a standard deviation of 1 to keep effects comparable across traits. All linear mixed-effects models were fitted using the lme function of the nlme package (Pinheiro et al., 2020) with the restricted maximum likelihood (REML) approach. Random effects were defined for each tree ID with random intercepts and random slopes for h_*i*_ and h_*i*_^2^ to account for individual growth patterns. The random effects structure was constructed with the pdDiag function from the nlme R-package. We accounted for first order autocorrelation in each tree using the corAR1 constructor from the nlme package. Weightings to correct for non-homogeneous variance between individuals were included with the varIdent function from the nlme package.

For pairwise comparison of site effects we used the lsmeans function of the lsmeans package with a Tukey-adjustment to account for non-homogeneous variances among the groups (Lenth, 2016).

**FIGURE 4:**
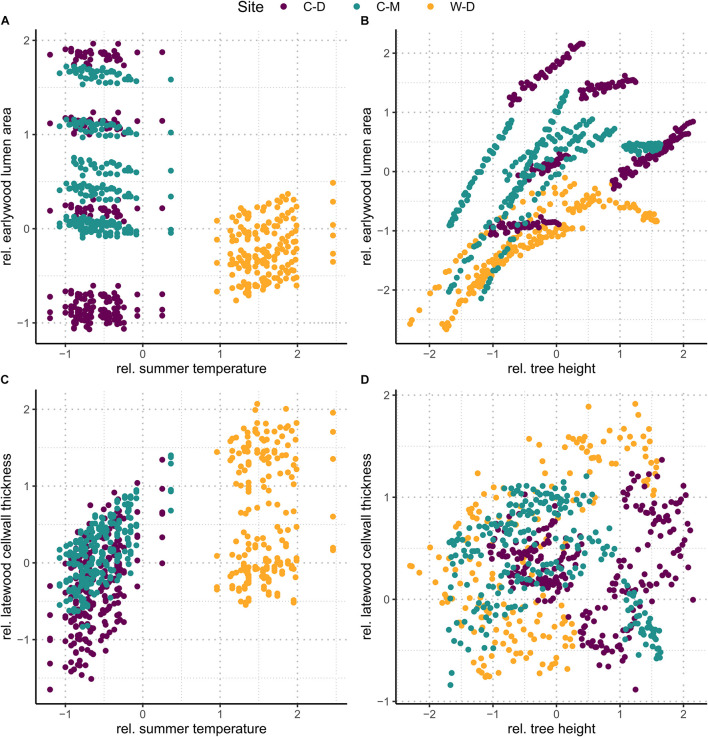
Upper row **(A,B)**: predicted relative earlywood lumen area. Lower row **(C,D)**: predicted relative latewood cell wall thickness. Left column **(A,C)**: values predicted on a dataset where height was set to the mean (i.e., 0). The figures show the effect of summer temperature without the effect of height. Right column **(B,D)**: values predicted on a dataset where summer temperature was set to the mean (i.e., 0). The figures show the effect of height without the effect of summer temperature.

To further visualize the effects of summer temperature and cumulative tree height increment, we used the linear mixed-effects model to predict trait values under two hypothetical conditions, i.e., (1) no change in tree height and (2) no change in summer temperature. First, we predicted trait values on a dataset, where tree height was set to the mean value (i.e., 0), while all other traits remained the same as in our original dataset to explore the effects of summer temperature (Figures 4A,C). Then we did the same on a second dataset, but instead of tree height, summer temperature was set to the mean value (i.e., 0) to explore the effects of tree height (Figures 4B,D).

## Results

Tree height reconstructions showed that trees from W-D on average grew faster in height and had a different allometry: they tended to grow taller when compared to trees from C-D or C-M of the same diameter (Figure 2). Trees from C-M grew faster than trees from C-D but they were similar in terms of allometry (Figure 2).

The linear mixed-effects models showed that the water transport-related traits LA and TB^2^ were mostly driven by tree height (Table 1 and Supplementary Table 2), especially in the earlywood. Specifically, LA was positively influenced by tree height in both earlywood and latewood with a particularly strong effect in earlywood (Table 1 and Figure 4B). Estimated climate effects on LA were only found for the reference site W-D (Table 1 and Figure 4A). Summer temperature showed a notable significant effect on earlywood lumen area at the W-D site (Table 1). Previous winter and spring precipitation had a positive effect on latewood LA at the W-D site (Table 1). TB^2^ was negatively influenced by tree height, both in earlywood and latewood, with stronger effects in the earlywood as well (Table 1). The most notable estimated climatic effects on TB^2^ were the negative effect of summer precipitation at the W-D site as well as the positive effect of summer temperature at the C-D and C-M sites (Table 1).

**TABLE 1:**
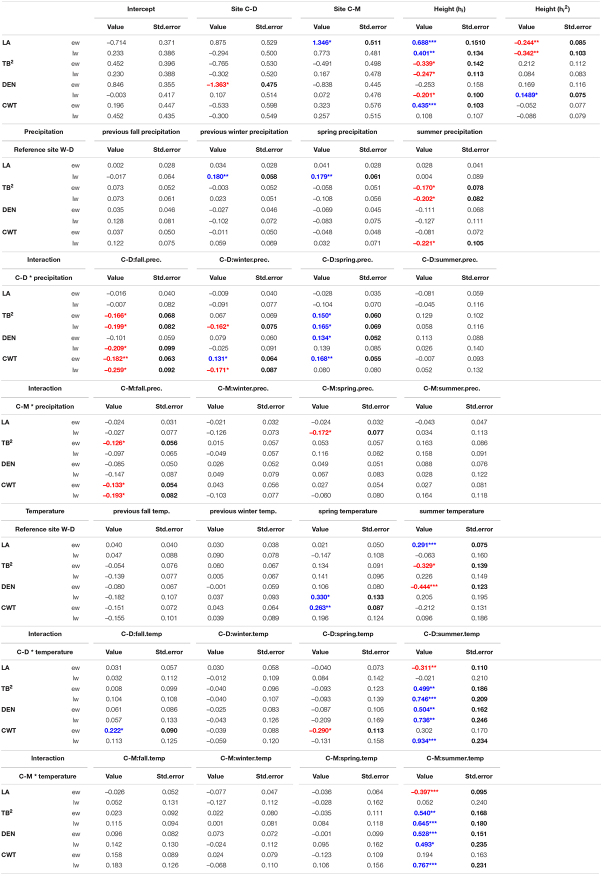
Results of trait specific linear mixed-effects models for the investigated traits (LA, lumen area**;** TB**^2^,** conduit reinforcement index**;** DEN, density**;** CWT, cell wall thickness) divided into earlywood (ew) and latewood (lw).

*Fixed effects: Intercept, site (C-D = Brooks Range, C-M = Denali NP) relative to Bluff (W-D), height, squared height, precipitation and temperature per season, interaction between seasonal precipitation/temperature and site, relative to Bluff (W-D), which was set as the reference point for the categorical variable “site.” Choosing another site as reference would not have changed the outcome of the models in a way that it would alter the interpretation of the results. Bold text shows significant values (p < 0.05 ^∗^p < 0.01 ^∗∗^p < 0.001 ^∗∗∗^, exact p-values are mentioned in Supplementary Table 2). Red text marks significant negative and blue marks significant positive effects.*

In contrast, DEN and CWT, which are related to structural integrity, were mainly influenced by climatic conditions, especially in the latewood. Summer temperature had a positive influence on DEN at the sites C-D and C-M, which was especially strong in the latewood. At W-D, latewood DEN was positively influenced by spring temperature (Table 1) and earlywood DEN was negatively influenced by summer temperature. Precipitation had only a weak influence on this trait (Table 1). DEN was only slightly negatively influenced by tree height in the latewood. CWT was most strongly impacted by summer temperature in the latewood at the sites C-D and C-M (Table 1 and Figure 4C). At W-D, latewood CWT was positively influenced by spring temperature and latewood CWT was negatively influenced by summer precipitation (Table 1). CWT was positively influenced by tree height in the earlywood.

**FIGURE 5:**
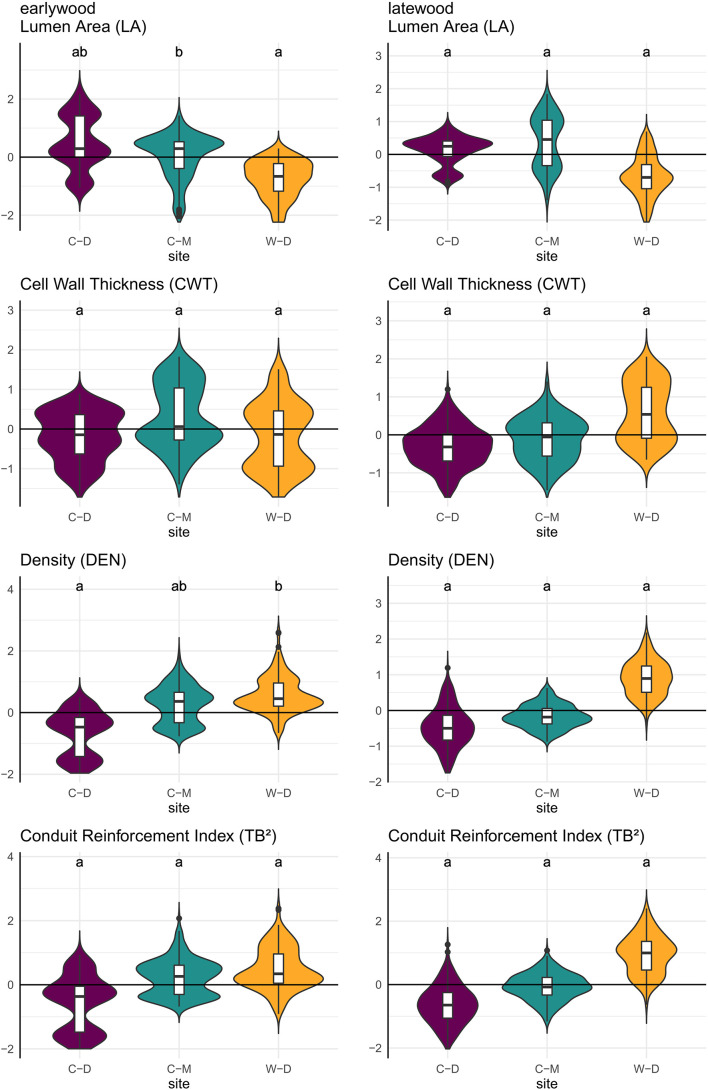
Violin plots combined with boxplots of predicted relative trait values (*y*-axis), based on linear mixed-effects models as described in the methods. Small letters indicate significance groups based on pairwise comparison with a significance threshold of *p* = 0.05. Purple = Brooks Range (C-D), Green = Denali National Park (C-M), Orange = Bluff (W-D).

The trait-values predicted by the linear mixed-effects models showed significant pairwise differences between C-M and W-D in predicted earlywood LA with W-D showing lower values, and between C-D and W-D in predicted earlywood density with W-D showing higher values (Figure 5 and Supplementary Table 3).

## Discussion

We used linear mixed-effects models to disentangle the effects of climatic conditions and tree height on four characteristic xylem anatomical traits. Our results can help to understand complex direct and indirect relationships between the investigated multifunctional xylem anatomical traits on the one hand and climatic conditions and physiological limitations on the other hand. We found that in earlywood, traits related to water transport (LA and TB^2^) were mainly driven by height at all sites, seemingly independent of the different climatic growth limitations (Figure 4). In the latewood, traits related to structural integrity (CWT and DEN) were mainly affected by climatic variables. We found a strong impact of summer temperature on latewood CWT (Table 1 and Figure 4C) especially in trees from the temperature-limited treeline sites C-D and C-M.

As expected, h_*i*_ had a strong effect, especially on traits related to water transport (LA and TB^2^), supporting our first hypothesis that earlywood traits related to water transport would be under a strong influence of tree height due to the importance of earlywood for water transport. Height influenced LA positively (i.e., larger lumen in taller trees) and TB^2^ negatively [i.e., taller trees have thinner cell walls relative to the cell wall length due to enlarged lumen with equal amounts of cell-wall material (Eq. 3)]. Similar results were found by Kašpar et al. (2019) for different conifer species at various elevational treelines. The strong positive effect of tree height on earlywood LA highlights the importance of “conduit widening” in conifers. Conduit widening describes the increase of LA from the apex toward the base of a tree. It is argued to occur due to the effects defined by the Hagen-Poiseuille law, which predicts a linearly increasing resistance with increasing conduit length for Newtonian fluids flowing in cylindrical conduits (Pfitzner, 1976; Gooch, 2011). This hydraulic resistance is maintained constant throughout the tree by conduit widening to ensure a cost-effective water transport (West et al., 1999; Becker et al., 2000; Enquist, 2002; Anfodillo et al., 2006). Consequently, when measuring LA from pith to bark at a particular height of the tree, it increases from year to year due to an increase of absolute tree height (Carrer et al., 2015). This effect also explains the influence of tree height on TB^2^. TB^2^ is the relation between CWT and cell wall length, and it increases with tree height because of the enlargement of cells while cell wall deposition stays similar. The usage of a constant amount of resources as mentioned in Cuny et al. (2014) leads to relatively thinner cell walls on bigger cells and thus a negative effect of tree height on TB^2^ (Hacke et al., 2001). As tree height itself is influenced by various environmental conditions, especially competition, temperature, and water availability (Lines et al., 2012; Fransson et al., 2021), it can be assumed that a large part of the height effects that we found correspond to indirect effects of environmental conditions on xylem anatomical traits related to water transport.

Regarding direct environmental effects, more complex patterns were found. LA did not seem to be strongly influenced by climatic conditions. Considering the rather weak effects of precipitation, that were only found in the latewood, the strong effects of tree height in earlywood and latewood (Table 1 and Figure 4) and the modest evidence for differences between sites in the earlywood lumen area (Figure 5), we can only assume that the potential impact of drought on LA was overruled by the height effect. Our third hypothesis, assuming smaller lumen area at the drought-limited W-D site as an adjustment for lowering the risk of cavitation, cannot be resolved, as evidence is deficient. The most noticeable climatic effect on LA was the positive effect of summer temperature on earlywood LA at W-D. Growth in general is positively correlated with temperature, thus warmer conditions could mean a greater increment in height and therefore bigger increase in lumen (Gillooly et al., 2001; Petit et al., 2011; Anfodillo et al., 2016). The height parameter used in the models is cumulative and might not fully reflect the effect of increased growth rates in warm years. Yet, the effect only occurred at W-D and cannot be explained with certainty.

Considering all sites together, the largest direct environmental effects on xylem anatomy were found in traits that are related to the structural integrity of the tree—DEN and CWT. With both latewood DEN and CWT being largely driven by temperature, our results support our second hypothesis stating that latewood traits related to structural integrity would be mainly influenced by climatic conditions. CWT was positively influenced by spring and summer temperature, yet this influence differed between sites. At the drought-limited site W-D only spring temperature showed a significantly positive but rather weak effect on earlywood CWT. However, as expected, we found a strong effect of summer temperature on latewood CWT for the sites C-D and C-M (Table 1 and Figure 4C). The increase of latewood CWT with increasing summer supports our fourth hypothesis stating that due to longer vegetation periods and higher temperatures CWT would be higher at W-D. Higher temperatures generally lead to faster biochemical processes and therefore a potentially faster cell wall deposition in trees, causing thicker cell walls (Yasue et al., 2000; Gillooly et al., 2001; Fonti et al., 2013; Rossi et al., 2014; Castagneri et al., 2017). Tree growth at the sites C-D and C-M is limited mainly by low temperatures, consequently leading to a high sensitivity to temperature. This effect is particularly apparent in latewood, toward the end of the vegetation period, since at the time of latewood formation it is more important for the tree to grow a stable wood structure with thick cell walls than to produce larger or more tracheids (Zobel and van Buijtenen, 1989; Fonti et al., 2013; Cuny and Rathgeber, 2016; Björklund et al., 2017). Additionally, we found a negative effect of summer precipitation on latewood CWT at the W-D and C-D sites. The increase in water availability could have induced an increase in the total lumen area while not influencing the total amount of cell wall material, leading to relatively thinner cell walls on larger or more tracheids (Cuny et al., 2014; Lange et al., 2020). Since in our study we did not find any noticeable effect of summer precipitation on LA, it is possible that higher water availability decreased CWT by increasing the number of cells instead of increasing their lumen area (Fonti et al., 2010; Jyske et al., 2010; Cuny and Rathgeber, 2016). We were not able to estimate the total number of cells per year, because it would have required a disproportionate amount of work to improve the sample quality further to a point where we would have been able to estimate a reliable result.

The effects found in DEN are in line with the effects found in CWT and LA. DEN is correlating with both CWT and LA due to the way it is calculated (Eq. 4). It must also be mentioned that according to the methodology of estimating density separately for earlywood and latewood it did not reflect the typical negative correlation to increased secondary growth induced by higher temperatures as found in other studies (Cortini et al., 2016; Hassegawa et al., 2019). DEN showed similar but overall relatively weaker reactions to climate conditions than CWT. This pattern might be explained by a stronger genetic control on DEN than on CWT, as found in previous studies (Lenz et al., 2011; Hassegawa et al., 2019; Pampuch et al., 2020). Therefore, it seems possible that especially the relation between total cell area and CWA (i.e., the anatomical allometry) is under a strong genetic control. Since TB^2^ is also derived from LA and CWT, climatic effects were found in TB^2^ as well. Again, these effects are in line with the effects we found in CWT. In contrast to DEN, earlywood TB^2^ expressed a slightly stronger response to summer precipitation and summer temperature than CWT. TB^2^ reflects the hydraulic safety, which is increased by building relatively thicker cell walls in relation to cell wall length (Hacke et al., 2001). This explains why latewood TB^2^ was negatively related to precipitation at the drought-limited site W-D (i.e., high precipitation leads to relatively bigger cell lumen).

Model predictions showed significant differences between the sites for earlywood LA and earlywood DEN (Figure 5). These differences must be interpreted carefully, as in both cases we had a high standard error and high *p*-values, indicating a weak confidence (Supplementary Table 2). Still, they showed that differences might also occur on yet another aspect that was not investigated in this study (e.g., adaptation; Lenz et al., 2011; Pampuch et al., 2020). Stronger differences between the sites regarding LA might have been mitigated by the beneficial effect of smaller lumen, which can also potentially reduce the risk of freeze-thaw embolisms (Pittermann and Sperry, 2006). However, a more reliable evidence for differences between the sites was expressed by the differences in allometry and growth speed (Figure 2), as well as by the significance and strength at which climatic drivers influenced the anatomy of trees (Table 1 and Figure 4). The differences in growth speed between the cold- and drought-limited sites were potentially caused by the differences in average temperatures and the length of the vegetation period (Figure 1). The higher temperatures and presumably longer vegetation period at W-D potentially led to longer and faster growth (Nienstaedt and Zasada, 1990; Gillooly et al., 2001) than at the other sites. The differences between the sites regarding the ratio between DBH and height, with relatively taller trees growing at W-D, was somewhat unexpected. Considering that W-D was classified as a drought-limited treeline and tree height influences the traits related to water transport, we expected trees to be rather short at W-D as a result of local phenotypic adjustment (Lines et al., 2012; Hacke et al., 2015; Stovall et al., 2019). It’s possible that in the sampled trees drought effects are not very pronounced due to their relatively short height, i.e., the trees might have not reached a critical size at which they would suffer severely from the dry conditions (Bennett et al., 2015). This could also explain why precipitation is not influencing LA. Yet, various other potential factors like competition for light (Grams and Andersen, 2007), differences in microsite-conditions (Li and Yang, 2004; Marquis et al., 2021; Zacharias et al., 2021), soil conditions (Urban et al., 2013) but also fire events (Johnstone et al., 2016; Whitman et al., 2019), or a combination of these factors could have had consequences for the forest dynamics and thus altered height growth and the allometry of white spruce at the W-D site. However, no detailed information on competition in the past, microsite or soil conditions was available to us to explain the found patterns with certainty.

## Conclusion

We found that, due to the multifunctionality of xylem tissue, anatomical traits are tied into complex multidimensional interactions between each other, direct and indirect climatic effects, and tree height.

Traits related to water transport, particularly earlywood lumen area, are indirectly influenced by the environmental conditions that affected tree height. Future studies aiming at examining xylem anatomical features of trees should be well aware of these effects, as they can heavily affect traits like LA when measured across an increment core. It is especially important to observe the correlation of tracheid dimensions with tree height when working on natural populations and on trees growing under varying environmental conditions.

Traits related to the structural integrity, particularly latewood CWT are under a strong direct influence of temperature. While not entirely new, this influence highlights the trait potential for dendroclimatological studies. With advancing technology, measuring xylem anatomical traits becomes less and less labor intensive. Latewood CWT could thus be used more widely for conducting studies on climate-growth correlations or to create climate reconstructions.

In general an increasing number of studies are taking advantage of investigating xylem anatomical traits. Yet, a proper interpretation of results requires a good level of understanding the function of xylem anatomical traits, how they are linked with each other, and to what extent they are influenced by environmental conditions. The complexity of xylem anatomy must be carefully considered, especially in studies that are conducted under natural conditions, and along gradients or in contrasting environments. Our study helps to better understand this complexity, and could be a useful inspiration for future studies to work on a more holistic approach to study xylem anatomy.

## Data Availability Statement

The raw data supporting the conclusions of this article will be made available by the authors, without undue reservation.

## Author Contributions

TP, MW, MT, and JL designed the study and conducted field work and sampling. TP, JL, and MT prepared the samples and performed xylem anatomical measurements. MT performed the statistical analysis for estimating site-specific tree height and stem diameter relationships. TP performed all further statistical analyses with help from AA-R. TP wrote the manuscript with contributions from all authors. All authors contributed to the article and approved the submitted version.

## Conflict of Interest

The authors declare that the research was conducted in the absence of any commercial or financial relationships that could be construed as a potential conflict of interest.

## Publisher’s Note

All claims expressed in this article are solely those of the authors and do not necessarily represent those of their affiliated organizations, or those of the publisher, the editors and the reviewers. Any product that may be evaluated in this article, or claim that may be made by its manufacturer, is not guaranteed or endorsed by the publisher.

## References

[B1] Abrahamson I. (2015). *Fire Effects Information System. Picea glauca.* Available online at: https://www.feis-crs.org/feis/ (accessed January 31, 2020)

[B2] Anfodillo T. Carraro V. Carrer M. Fior C. Rossi S. (2006). Convergent tapering of xylem conduits in different woody species. *New Phytol.* 169 279–290. 10.1111/j.1469-8137.2005.01587.x 16411931

[B3] Anfodillo T. Petit G. Crivellaro A. (2013). Axial conduit widening in woody species: a still neglected anatomical pattern. *IAWA J.* 34 352–364. 10.1163/22941932-00000030

[B4] Anfodillo T. Petit G. Sterck F. Lechthaler S. Olson M. E. (2016). Allometric trajectories and “stress”: A quantitative approach. *Front. Plant Sci.* 7:1681. 10.3389/fpls.2016.01681 27881990 PMC5101416

[B5] Antonova G. F. Stasova V. V. (1993). Effects of environmental factors on wood formation in Scots pine stems. *Trees* 7 214–219. 10.1007/BF00202076

[B6] Arneth A. Harrison S. P. Zaehle S. Tsigaridis K. Menon S. Bartlein P. J. (2010). Terrestrial biogeochemical feedbacks in the climate system. *Nat. Geosci.* 3 525–532. 10.1038/ngeo905

[B7] Attree S. M. Dunstan D. I. Fowke L. C. (1991). *Trees III.* (Berlin: Springer)

[B8] Barber V. Juday G. Finney B. (2000). Reduced growth of Alaska white spruce in the twentieth century from temperature-induced drought stress. *Nature* 405 668–672. 10864320 10.1038/35015049

[B9] Becker P. Gribben R. J. Lim C. M. (2000). Tapered conduits can buffer hydraulic conductance from path-length effects. *Tree Physiol.* 20 965–967. 10.1093/treephys/20.14.965 11303571

[B10] Bennett A. C. Mcdowell N. G. Allen C. D. Anderson-Teixeira K. J. (2015). Larger trees suffer most during drought in forests worldwide. *Nat. Plants* 1:139. 10.1038/nplants.2015.139 27251391

[B11] Bieniek P. A. Bhatt U. S. Walsh J. E. Rupp T. S. Zhang J. Krieger J. R. (2016). Full access dynamical downscaling of ERA-interim temperature and precipitation for Alaska. *J. Appl. Meteorol. Climatol.* 55 635–654. 10.1175/JAMC-D-15-0153.1

[B12] Björklund J. Seftigen K. Fonti P. Nievergelt D. von Arx G. (2020). Dendroclimatic potential of dendroanatomy in temperature-sensitive Pinus sylvestris. *Dendrochronologia* 60:125673. 10.1016/j.dendro.2020.125673

[B13] Björklund J. Seftigen K. Schweingruber F. Fonti P. von Arx G. Bryukhanova M. V. (2017). Cell size and wall dimensions drive distinct variability of earlywood and latewood density in Northern Hemisphere conifers. *New Phytol.* 216 728–740. 10.1111/nph.14639 28636081

[B14] Brockmann-Jerosch H. (1919). *Baumgrenze und Klimacharakter. Pflanzengeographische Kommission der Schweizerischen Naturforschenden Gesellschaft. Beiträge zur geobotanischen Landesaufnahme, Bd. 6.* Zürich: Rascher & Cie.

[B15] Bunn A. G. (2008). A dendrochronology program library in R (dplR). *Dendrochronologia* 26 115–124. 10.1016/j.dendro.2008.01.002

[B16] Callaham R. Z. (1962). Geographic variability in growth of forest trees. *Tree Growth* 1962 311–325.

[B17] Carrer M. von Arx G. Castagneri D. Petit G. (2015). Distilling allometric and environmental information from time series of conduit size: the standardization issue and its relationship to tree hydraulic architecture. *Tree Physiol.* 35 27–33. 10.1093/treephys/tpu108 25576756

[B18] Castagneri D. Fonti P. Von Arx G. Carrer M. (2017). How does climate influence xylem morphogenesis over the growing season? Insights from long-Term intra-ring anatomy in Picea abies. *Anna. Bot.* 119 1011–1020. 10.1093/aob/mcw274 28130220 PMC5604563

[B19] Charney N. D. Babst F. Poulter B. Record S. Trouet V. M. Frank D. (2016). Observed forest sensitivity to climate implies large changes in 21st century North American forest growth. *Ecol. Lett.* 19 1119–1128. 10.1111/ele.12650 27434040

[B20] Cochard H. (2006). Cavitation in trees. *Comptes Rendus Physique* 7 1018–1026. 10.1016/j.crhy.2006.10.012

[B21] Cortini F. MacIsaac D. Comeau P. (2016). White spruce growth and wood properties over multiple time periods in relation to current tree and stand attributes. *Forests* 7:49. 10.3390/f7030049

[B22] Cuny H. E. Fonti P. Rathgeber C. B. K. von Arx G. Peters R. L. Frank D. C. (2019). Couplings in cell differentiation kinetics mitigate air temperature influence on conifer wood anatomy. *Plant Cell Environ.* 42 1222–1232. 10.1111/pce.13464 30326549

[B23] Cuny H. E. Rathgeber C. B. K. (2016). Xylogenesis: Coniferous trees of temperate forests are listening to the climate tale during the growing season but only remember the last words! Plant Physiology. 171 306–317. 10.1104/pp.16.00037 27208048 PMC4854703

[B24] Cuny H. E. Rathgeber C. B. K. Frank D. Fonti P. Fournier M. (2014). Kinetics of tracheid development explain conifer tree-ring structure. *New Phytol.* 203 1231–1241. 10.1111/nph.12871 24890661

[B25] Däniker A. (1932). *Biologische Studien über Baum- und Waldgrenze, insbesondere über die klimatischen Ursachen und deren Zusammenhänge.* Zürich: Vierteljahrsschrift d. Naturf. Ges.

[B26] Denne M. P. (1989). Definition of Latewood According to Mork (1928). *IAWA J.* 10 59–62. 10.1163/22941932-90001112

[B27] Domec J.-C. Gartner B. L. (2002). How do water transport and water storage differ in coniferous earlywood and latewood? *J. Experimen. Bot.* 53 2369–2379. 10.1093/jxb/erf100 12432029

[B28] Eldhuset T. D. Nagy N. E. Volařík D. Børja I. Gebauer R. Yakovlev I. A. (2013). Drought affects tracheid structure, dehydrin expression, and above- and belowground growth in 5-year-old Norway spruce. *Plant Soil* 366 305–320. 10.1007/s11104-012-1432-z

[B29] Enquist B. J. (2002). Universal scaling in tree and vascular plant allometry: Toward a general quantitative theory linking plant form and function from cells to ecosystems. *Tree Physiol.* 22 1045–1064. 10.1093/treephys/22.15-16.1045 12414366

[B30] Eusemann P. Schnittler M. Nilsson R. H. Jumpponen A. Dahl M. B. Würth D. G. (2016). Habitat conditions and phenological tree traits overrule the influence of tree genotype in the needle mycobiome–Picea glauca system at an arctic treeline ecotone. *New Phytol.* 211 1221–1231. 10.1111/nph.13988 27144386

[B31] Fonti P. Bryukhanova M. V. Myglan V. S. Kirdyanov A. V. Naumova O. V. Vaganov E. A. (2013). Temperature-induced responses of xylem structure of Larix sibirica (pinaceae) from the Russian Altay. *Am. J. Bot.* 100 1332–1343. 10.3732/ajb.1200484 23660567

[B32] Fonti P. von Arx G. García-González I. Eilmann B. Sass-Klaassen U. Gärtner H. (2010). Studying global change through investigation of the plastic responses of xylem anatomy in tree rings. *New Phytol.* 185 42–53. 10.1111/j.1469-8137.2009.03030.x 19780986

[B33] Fransson P. Brännström A. Franklin O. (2021). A tree’s quest for light-optimal height and diameter growth under a shading canopy. *Tree Physiol.* 41 1–11. 10.1093/treephys/tpaa110 32879970 PMC7868666

[B34] Gärtner H. Nievergelt D. (2010). The core-microtome: A new tool for surface preparation on cores and time series analysis of varying cell parameters. *Dendrochronologia* 28 85–92. 10.1016/j.dendro.2009.09.002

[B35] Gärtner H. Schweingruber F. H. (2013). *Microscopic preparation techniques for plant stem analysis.* Remagen: Verlag Dr. Kessel.

[B36] Gauthier S. Bernier P. Kuuluvainen T. Shvidenko A. Z. Schepaschenko D. G. (2015). Boreal forest health and global change. *Science* 349 819–822. 10.1126/science.aaa9092 26293953

[B37] Gillooly J. F. Brown J. H. West G. B. Savage V. M. Charnov E. L. (2001). Effects of size and temperature on metabolic rate. *Science* 293 2248–2251. 10.1126/science.1061967 11567137

[B38] Gooch J. W. (2011). “Hagen-Poiseuille Equation,” in *Encyclopedic Dictionary of Polymers*, ed. Gooch J. W. (New York, NY: Springer).

[B39] Grace J. Berninger F. Nagy L. (2002). Impacts of climate change on the tree line. *Anna. Bot.* 90 537–544. 10.1093/aob/mcf222 12324278 PMC4240388

[B40] Grams T. E. E. Andersen C. P. (2007). *Competition for resources in trees: physiological versus morphological plasticity in progress in botany.* Berlin: Springer. 356–381. 10.1007/978-3-540-36832-8_16

[B41] Grothendieck G. (2013). nls2: Non-linear regression with brute force. Available online at: https://cran.r-project.org/package=nls2 (accessed March 08, 2013)

[B42] Hacke U. G. Lachenbruch B. Pittermann J. Mayr S. Domec J.-C. Schulte P. J. (2015). *Functional and Ecological Xylem Anatomy.* (Berlin: Springer International Publishing), 10.1007/978-3-319-15783-2

[B43] Hacke U. G. Sperry J. S. (2001). Functional and ecological xylem anatomy. *Pers. Plant Ecol. Evol. Syst.* 4 97–115. 10.1078/1433-8319-00017

[B44] Hacke U. G. Sperry J. S. Pockman W. T. Davis S. D. McCulloh K. A. (2001). Trends in wood density and structure are linked to prevention of xylem implosion by negative pressure. *Oecologia* 126 457–461. 10.1007/s004420100628 28547229

[B45] Hassegawa M. Savard M. Lenz P. R. N. Duchateau E. Gélinas N. Bousquet J. (2019). White spruce wood quality for lumber products: priority traits and their enhancement through tree improvement. *Forestry* 93 1–22. 10.1093/forestry/cpz050

[B46] Huang S. Titus S. J. Wiens D. P. (1992). Comparison of nonlinear height–diameter functions for major Alberta tree species. *Can. J. Forest Res.* 22 1297–1304. 10.1139/x92-172 33356898

[B47] IPCC. (2021). “Climate Change 2021: The Physical Science Basis”. in *Contribution of Working Group I to the Sixth Assessment Report of the Intergovernmental Panel on Climate Change*, eds MassonDelmotte V. Zhai P. Pirani A. Connors S. L. Pean C. Berger S. (Cambridge: Cambridge University Press).

[B48] Johnstone J. F. Allen C. D. Franklin J. F. Frelich L. E. Harvey B. J. Higuera P. E. (2016). Changing disturbance regimes, ecological memory, and forest resilience. *Front. Ecol. Environ.* 14:369–378. 10.1002/fee.1311

[B49] Juday G. P. Barber V. Rupp S. Zasada J. Wilmking M. (2003). *A 200-Year perspective of climate variability and the response of white spruce in interior alaska. in Climate Variability and Ecosystem Response at Long-Term Ecological Research Sites.* Oxford: Oxford University Press.226–250.

[B50] Jyske T. Holtta T. Makinen H. Nojd P. Lumme I. Spiecker H. (2010). The effect of artificially induced drought on radial increment and wood properties of Norway spruce. *Tree Physiol.* 30 103–115. 10.1093/treephys/tpp099 19955191

[B51] Kašpar J. Anfodillo T. Treml V. (2019). Tree size mostly drives the variation of xylem traits at the treeline ecotone. *Trees Struct. Funct.* 33 1657–1665. 10.1007/s00468-019-01887-6

[B52] Körner C. (2007). Climatic Treelines: Conventions. Global Patterns, Causes (Klimatische Baumgrenzen: Konventionen, globale Muster, Ursachen). *Erdkunde* 4 316–324.

[B53] Lange J. Carrer M. Pisaric M. F. J. Porter T. J. Seo J. Trouillier M. (2020). Moisture−driven shift in the climate sensitivity of white spruce xylem anatomical traits is coupled to large−scale oscillation patterns across northern treeline in northwest North America. *Glob. Change Biol.* 26 1842–1856. 10.1111/gcb.14947 31799729

[B54] Lenth R. V. (2016). Least-Squares Means: The R Package lsmeans. *J. Statist. Softw.* 69 1–33. 10.18637/jss.v069.i01

[B55] Lenz P. MacKay J. Rainville A. Cloutier A. Beaulieu J. (2011). The influence of cambial age on breeding for wood properties in Picea glauca. *Tree Genet. Genomes* 7 641–653. 10.1007/s11295-011-0364-8

[B56] Li M.-H. Yang J. (2004). Effects of microsite on growth of Pinus cembra in the subalpine zone of the Austrian Alps. *Anna. Forest Sci.* 61 319–325. 10.1051/forest:2004025

[B57] Lines E. R. Zavala M. A. Purves D. W. Coomes D. A. (2012). Predictable changes in aboveground allometry of trees along gradients of temperature, aridity and competition. *Glob. Ecol Biogeography* 21 1017–1028. 10.1111/j.1466-8238.2011.00746.x

[B58] Little E. L. Viereck L. A. (1971). *Atlas of United States trees.* Washington, DC: U.S. Dept. of Agriculture, Forest Service

[B59] Lloyd A. H. Duffy P. A. Mann D. H. (2013). Nonlinear responses of white spruce growth to climate variability in interior Alaska. *Can. J. Forest Res.* 43 331–343. 10.1139/cjfr-2012-0372 33356898

[B60] Lloyd A. H. Fastie C. L. (2002). Spatial and temporal variability in the growth and climate response of treeline trees in Alaska. *Clim. change* 52 481–509. 10.1023/a:1014278819094

[B61] Marquis B. Duval P. Bergeron Y. Simard M. Thiffault N. Tremblay F. (2021). Height growth stagnation of planted spruce in boreal mixedwoods: Importance of landscape, microsite, and growing-season frosts. *Forest Ecol. Manag.* 479:118533. 10.1016/j.foreco.2020.118533

[B62] Mencuccini M. Hölttä T. Petit G. Magnani F. (2007). Sanio’s laws revisited. Size-dependent changes in the xylem architecture of trees. *Ecol. Lett.* 10 1084–1093. 10.1111/j.1461-0248.2007.01104.x 17850336

[B63] Myburg A. A. Lev-Yadun S. Sederoff R. R. (2013). Xylem Structure and Function. *eLS* 2013 1–19. 10.1002/9780470015902.a0001302.pub2

[B64] Nienstaedt H. Zasada J. C. (1990). Picea glauca (Moench) Voss white spruce. *Silvics North Am.* 1 204–226.

[B65] Pampuch T. Anadon-Rosell A. Zacharias M. von Arx G. Wilmking M. (2020). Xylem anatomical variability in white spruce at treeline is largely driven by spatial clustering. *Front. Plant Sci.* 11:1–10. 10.3389/fpls.2020.581378 33193527 PMC7609655

[B66] Petit G. Anfodillo T. Carraro V. Grani F. Carrer M. (2011). Hydraulic constraints limit height growth in trees at high altitude. *New Phytol.* 189 241–252. 10.1111/j.1469-8137.2010.03455.x 20840508

[B67] Pfitzner J. (1976). Poiseuille and his law. *Anaesthesia* 31 273–275. 10.1111/j.1365-2044.1976.tb11804.x 779509

[B68] Pinheiro J. Bates D. DebRoy S. Sarkar D. R Core Team. (2020). *{nlme}: Linear and Nonlinear Mixed Effects Models.* Available online at: https://cran.r-project.org/package=nlme (accessed September 7, 2021)

[B69] Pittermann J. Sperry J. S. (2006). Analysis of freeze-thaw embolism in conifers. The interaction between cavitation pressure and tracheid size. *Plant Physiol.* 140 374–382. 10.1104/pp.105.067900 16377751 PMC1326058

[B70] Pittermann J. Sperry J. S. Wheeler J. K. Hacke U. G. Sikkema E. H. (2006). Mechanical reinforcement of tracheids compromises the hydraulic efficiency of conifer xylem. *Plant Cell Environ.* 29 1618–1628. 10.1111/j.1365-3040.2006.01539.x 16898022

[B71] Prendin A. L. Petit G. Carrer M. Fonti P. Björklund J. Von Arx G. (2017). New research perspectives from a novel approach to quantify tracheid wall thickness. *Tree Physiol.* 37 1–8. 10.1093/treephys/tpx037 28379577

[B72] Puchi P. F. Castagneri D. Rossi S. Carrer M. (2020). Wood anatomical traits in black spruce reveal latent water constraints on the boreal forest. *Glob. Change Biol.* 26 1767–1777. 10.1111/gcb.14906 31692158

[B73] R Core Team (2020). *R: A language and environment for statistical computing.* Vienna: R Core Team.

[B74] Rossi S. Girard M. J. Morin H. (2014). Lengthening of the duration of xylogenesis engenders disproportionate increases in xylem production. *Glob. Change Biol.* 20 2261–2271. 10.1111/gcb.12470 24259354

[B75] Sanio K. (1872). Ueber die Grösse der Holzzellen bei der gemeinen Kiefer (Pinus silvestris). *Jahrbücher für wissenschaftliche Botanik.* 8 401–420.

[B76] Sherriff R. L. Miller A. E. Muth K. Schriver M. Batzel R. (2017). Spruce growth responses to warming vary by ecoregion and ecosystem type near the forest-tundra boundary in south-west Alaska. *J. Biogeogra.* 44 1457–1468. 10.1111/jbi.12968

[B77] SNAP. (2019). *Scenarios network for Alaska and arctic planning.* Available online at: http://ckan.snap.uaf.edu/dataset. (accessed June 23, 2019)

[B78] Soja A. J. Tchebakova N. M. French N. H. F. Flannigan M. D. Shugart H. H. Stocks B. J. (2007). Climate-induced boreal forest change: Predictions versus current observations. *Glob. Planet. Change* 56 274–296. 10.1016/j.gloplacha.2006.07.028

[B79] Stovall A. E. L. Shugart H. Yang X. (2019). Tree height explains mortality risk during an intense drought. *Nat. Commun.* 10:4385. 10.1038/s41467-019-12380-6 31558795 PMC6763443

[B80] Tagesson T. Schurgers G. Horion S. Ciais P. Tian F. Brandt M. (2020). Recent divergence in the contributions of tropical and boreal forests to the terrestrial carbon sink. *Nat. Ecol. Evol.* 4 202–209. 10.1038/s41559-019-1090-0 31988446

[B81] Trouillier M. van der Maaten-Theunissen M. Harvey J. Würth D. Schnittler M. Wilmking M. (2018). Visualizing individual tree differences in tree-ring studies. *Forests* 9:216. 10.3390/f9040216

[B82] Trouillier M. van der Maaten-Theunissen M. Scharnweber T. Würth D. Burger A. Schnittler M. (2019). Size matters—a comparison of three methods to assess age- and size-dependent climate sensitivity of trees. *Trees Struct. Funct.* 33 183–192. 10.1007/s00468-018-1767-z

[B83] Ueyama M. Iwata H. Harazono Y. Euskirchen E. S. Oechel W. C. Zona D. (2013). Growing season and spatial variations of carbon fluxes of Arctic and boreal ecosystems in Alaska (USA). *Ecol. Appl.* 23 1798–1816. 10.1890/11-0875.1 24555310

[B84] Urban J. Holušová K. Menšík L. Čermák J. Kantor P. (2013). Tree allometry of Douglas fir and Norway spruce on a nutrient-poor and a nutrient-rich site. *Trees Struct. Funct.* 27 97–110. 10.1007/s00468-012-0771-y

[B85] von Arx G. Carrer M. (2014). Roxas -A new tool to build centuries-long tracheid-lumen chronologies in conifers. *Dendrochronologia* 32 290–293. 10.1016/j.dendro.2013.12.001

[B86] von Arx G. Crivellaro A. Prendin A. L. Čufar K. Carrer M. (2016). Quantitative Wood Anatomy—Practical Guidelines. *Front. Plant Sci.* 7:1–13. 10.3389/fpls.2016.00781 27375641 PMC4891576

[B87] West G. B. Brown J. H. Enquist B. J. (1999). A general model for the structure and allometry of plant vascular systems. *Nature* 400 664–667. 10.1038/23251

[B88] Whitman E. Parisien M. A. Thompson D. K. Flannigan M. D. (2019). Short-interval wildfire and drought overwhelm boreal forest resilience. *Sci. Rep.* 9 1–12. 10.1038/s41598-019-55036-7 31827128 PMC6906309

[B89] Wieser G. (2020). Alpine and polar treelines in a changing environment. *Forests* 11:254. 10.3390/f11030254

[B90] Wilmking M. Buras A. Eusemann P. Schnittler M. Trouillier M. Würth D. (2017). High frequency growth variability of White spruce clones does not differ from non-clonal trees at Alaskan treelines. *Dendrochronologia* 44 187–192. 10.1016/j.dendro.2017.05.005

[B91] Wilmking M. Juday G. P. (2005). Longitudinal variation of radial growth at Alaska’s northern treeline - Recent changes and possible scenarios for the 21st century. *Glob. Planet. Change* 47 282–300. 10.1016/j.gloplacha.2004.10.017

[B92] Yasue K. Funada R. Kobayashi O. Ohtani J. (2000). The effects of tracheid dimensions on variations in maximum density of Picea glehnii and relationships to climatic factors. *Trees Struct. Funct.* 14 223–229. 10.1007/PL00009766

[B93] Zacharias M. Pampuch T. Heer K. Avanzi C. Würth D. G. Trouillier M. (2021). Population structure and the influence of microenvironment and genetic similarity on individual growth at Alaskan white spruce treelines. *Sci. Total Environ.* 798:149267. 10.1016/j.scitotenv.2021.149267 34332391

[B94] Zobel B. J. van Buijtenen J. P. (1989). *Wood Variation and Wood Properties.* Berlin: Springer. 10.1007/978-3-642-74069-5_1

