# Supplementary material for: Direct and Indirect Effects of Environmental Limitations on White Spruce Xylem Anatomy at Treeline
Source: Front Plant Sci. 2021 Oct 25;12:748055. doi: 10.3389/fpls.2021.748055 (PMC8573320; doi:10.3389/fpls.2021.748055)
Supplement: Supplementary file 1 [file Data_Sheet_1.docx]

Direct and indirect effects of environmental limitations on white spruce xylem anatomy at treeline

Supplementary material


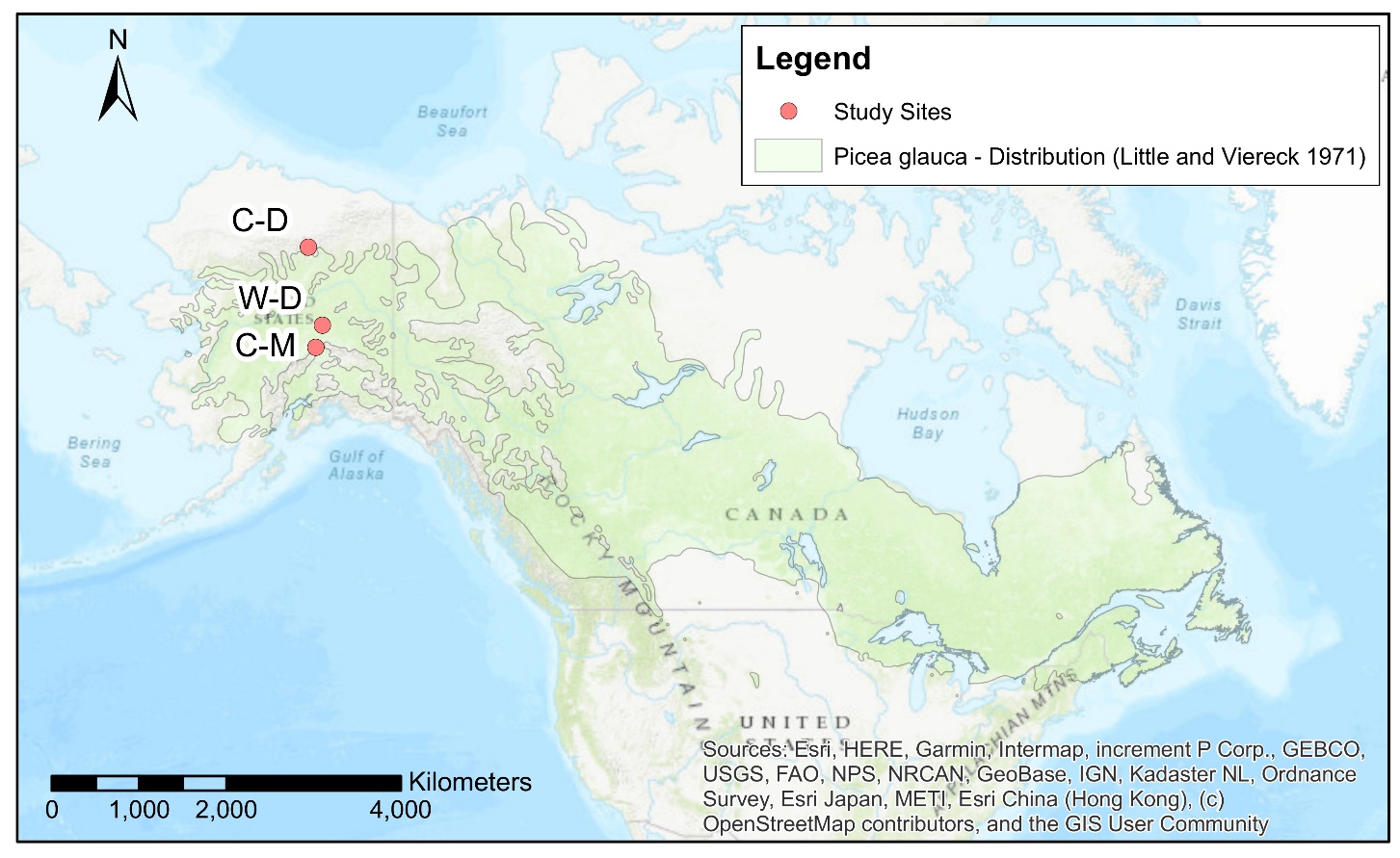


**Figure S1. Map of Northern America with study sites** (red dots; C-D = Brooks Range, C-M = Denali National Park, W-D = Bluff) and distribution of Picea glauca (green colored area) according to Little and Viereck 1971.


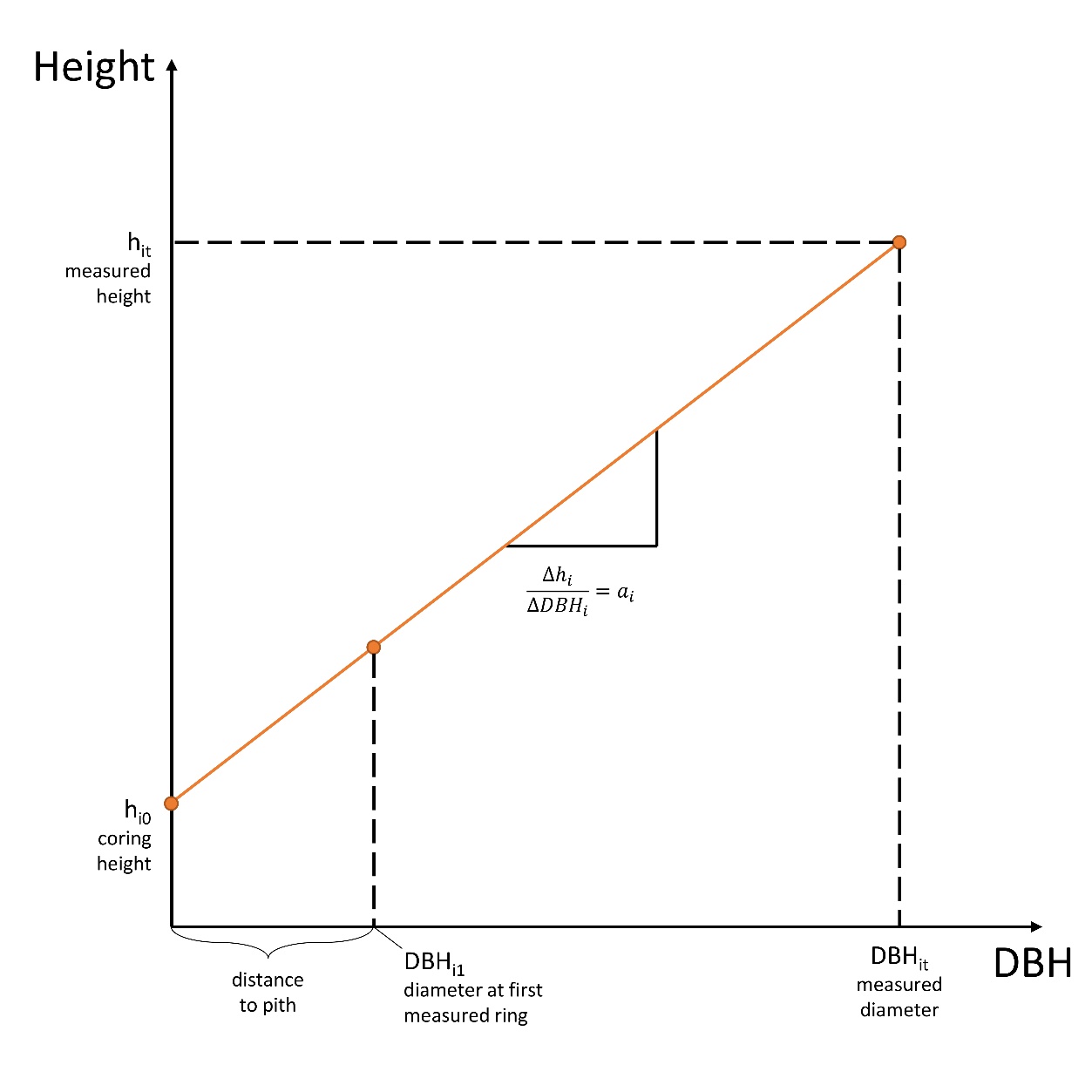


**Figure S2. Linear relation between tree height and DBH as assumed for calculating tree height in equation 4.**

**Table S1. Individual tree parameters**

| Tree ID | Study Site | Height (m) | DBH (cm) | Coring Height (cm) |
| --- | --- | --- | --- | --- |
| 45210a | W-D | 5.6 | 9 | 35 |
| 45228a | W-D | 5.5 | 8.5 | 40 |
| 45239a | W-D | 6.4 | 9.5 | 30 |
| 45261a | W-D | 6.1 | 9.7 | 40 |
| 45264a | W-D | 5.4 | 9.5 | 40 |
| 45393 | W-D | 6.9 | 10.1 | 30 |
| 254 | C-D | 4.8 | 9.5 | 37 |
| 283 | C-D | 6.4 | 17.5 | 50 |
| 299 | C-D | 5.3 | 28 | 34 |
| 54 | C-D | 7.6 | 15.5 | 75 |
| 91 | C-D | 5 | 9 | 20 |
| 94 | C-D | 7.5 | 13 | 32 |
| PE460a | C-M | 7 | 23.5 | 95 |
| PE461a | C-M | 5.7 | 11 | 105 |
| PE462b | C-M | 6.2 | 15.5 | 125 |
| PE467.2a | C-M | 4 | 9 | 100 |
| PE467b | C-M | 5.8 | 16 | 120 |
| PE470b | C-M | 5.2 | 13 | 110 |

**Table S2 p-values of Table 1**

|  |  | intercept | site C-D | site C-M | height (h_i_) | height (h_i_ ²) |
| --- | --- | --- | --- | --- | --- | --- |
|  |  | p | p | p | p | p |
| LA | ew | 0.0548 | 0.1189 | **0.0187*** | **6.12E-06***** | **0.0043**** |
|  | lw | 0.5464 | 0.5656 | 0.1291 | **0.0028**** | **0.0010**** |
| TB² | ew | 0.2546 | 0.1693 | 0.3394 | **0.0175*** | 0.0587 |
|  | lw | 0.5538 | 0.5700 | 0.7317 | **0.0290*** | 0.3074 |
| DEN | ew | 0.0174 | **0.0118*** | 0.0795 | 0.1099 | 0.1464 |
|  | lw | 0.9938 | 0.8385 | 0.8821 | **0.0448*** | **0.0473*** |
| CWT | ew | 0.6609 | 0.3867 | 0.5836 | **3.00E-05***** | 0.5028 |
|  | lw | 0.2992 | 0.5921 | 0.6251 | 0.3114 | 0.2760 |

| Precipitation | | previous fall precipitation | previous winter precipitation | spring precipitation | summer precipitation |
| --- | --- | --- | --- | --- | --- |
| Reference site W-D | | p | p | p | p |
| LA | ew | 0.9466 | 0.2296 | 0.1472 | 0.4957 |
|  | lw | 0.7951 | **0.0021**** | **0.0034**** | 0.9668 |
| TB² | ew | 0.1615 | 0.9487 | 0.2577 | **0.0295*** |
|  | lw | 0.2281 | 0.6495 | 0.0541 | **0.0137*** |
| DEN | ew | 0.4525 | 0.5669 | 0.1265 | 0.1056 |
|  | lw | 0.1131 | 0.1542 | 0.2681 | 0.2524 |
| CWT | ew | 0.4558 | 0.8301 | 0.3210 | 0.2612 |
|  | lw | 0.1056 | 0.3918 | 0.6487 | **0.0366*** |
| Temperature | | **previous fall temp.** | **previous winter temp.** | **spring temperature** | **summer temperature** |
| Reference site W-D | | p | p | p | p |
| LA | ew | 0.3229 | 0.4236 | 0.6739 | **0.0001***** |
|  | lw | 0.5903 | 0.2502 | 0.1748 | 0.6918 |
| TB² | ew | 0.4786 | 0.3663 | 0.1417 | **0.0181*** |
|  | lw | 0.0702 | 0.9445 | 0.1403 | 0.1290 |
| DEN | ew | 0.2313 | 0.9921 | 0.1876 | **0.0004***** |
|  | lw | 0.0904 | 0.6907 | **0.01352*** | 0.2921 |
| CWT | ew | 0.0357 | 0.5016 | **0.0026**** | 0.1070 |
|  | lw | 0.1240 | 0.6576 | 0.1159 | 0.6065 |
| Interaction | | **C-D:fall prec.** | **C-D:winter prec.** | **C-D:spring prec.** | **C-D:summer prec.** |
| C-D * precipitation | | p | p | p | p |
| LA | ew | 0.6815 | 0.8308 | 0.4171 | 0.1692 |
|  | lw | 0.9361 | 0.2336 | 0.1417 | 0.6980 |
| TB² | ew | **0.0154*** | 0.3257 | **0.0128*** | 0.2091 |
|  | lw | **0.01561*** | **0.0306*** | **0.0166*** | 0.6172 |
| DEN | ew | 0.0878 | 0.1862 | **0.0111*** | 0.2000 |
|  | lw | **0.0360*** | 0.7838 | 0.1028 | 0.8521 |
| CWT | ew | **0.0038**** | **0.0402*** | **0.0025**** | 0.9373 |
|  | lw | **0.0049**** | **0.0486*** | 0.3206 | 0.6912 |

| Interaction | | C-M:fall prec. | C-M:winter prec. | C-M:spring prec. | C-M:summer prec. |
| --- | --- | --- | --- | --- | --- |
| C-M * precipitation | | p | p | p | p |
| LA | ew | 0.4433 | 0.5195 | 0.4480 | 0.3619 |
|  | lw | 0.7279 | 0.0829 | **0.0261*** | 0.7633 |
| TB² | ew | **0.0255*** | 0.7961 | 0.3461 | 0.0580 |
|  | lw | 0.1356 | 0.3878 | 0.0630 | 0.0836 |
| DEN | ew | 0.0900 | 0.6120 | 0.3292 | 0.2457 |
|  | lw | 0.0910 | 0.5337 | 0.4192 | 0.8208 |
| CWT | ew | **0.0148*** | 0.4480 | 0.6232 | 0.7423 |
|  | lw | **0.0184*** | 0.1821 | 0.4537 | 0.1650 |
| Interaction | | **C-D:fall temp.** | **C-D:winter temp.** | **C-D:spring temp.** | **C-D:summer temp.** |
| C-D * temperature | | p | p | p | p |
| LA | ew | 0.5882 | 0.6113 | 0.5808 | **0.0047**** |
|  | lw | 0.7767 | 0.9150 | 0.5560 | 0.9209 |
| TB² | ew | 0.9327 | 0.6790 | 0.4461 | **0.0075**** |
|  | lw | 0.3386 | 0.7157 | 0.5029 | **0.0004***** |
| DEN | ew | 0.4736 | 0.7634 | 0.4128 | **0.0020**** |
|  | lw | 0.6702 | 0.7359 | 0.2151 | **0.0029**** |
| CWT | ew | **0.0141*** | 0.6605 | **0.0103*** | 0.0762 |
|  | lw | 0.3666 | 0.6214 | 0.4070 | **8.00E-05***** |
| Interaction | | **C-M:fall temp.** | **C-M:winter temp.** | **C-M:spring temp.** | **C-M:summer temp.** |
| C-M * temperature | | p | p | p | p |
| LA | ew | 0.6193 | 0.1035 | 0.5729 | **3.59E-05***** |
|  | lw | 0.6930 | 0.2565 | 0.8648 | 0.8282 |
| TB² | ew | 0.8059 | 0.7854 | 0.7524 | **0.0013**** |
|  | lw | 0.2220 | 0.9954 | 0.4770 | **0.0004***** |
| DEN | ew | 0.2403 | 0.3153 | 0.9951 | **0.0005***** |
|  | lw | 0.2753 | 0.8323 | 0.5574 | **0.0364*** |
| CWT | ew | 0.0767 | 0.7619 | 0.2568 | 0.2351 |
|  | lw | 0.1470 | 0.5337 | 0.4990 | **0.0009***** |

**Table S3. Linear mixed-effects models specific marginal R-squared (R²_m_) and conditional R-squared (R²_c_)**

| Trait | | R²_m_ (%) | R²_c_ (%) |
| --- | --- | --- | --- |
| LA | Earlywood | 35 | 96 |
|  | Latewood | 38 | 87 |
| TB² | Earlywood | 18 | 79 |
|  | Latewood | 32 | 76 |
| CWT | Earlywood | 21 | 86 |
|  | Latewood | 28 | 81 |
| DEN | Earlywood | 21 | 81 |
|  | Latewood | 38 | 75 |

**Table S4. Results of the pairwise comparison of site effects**

|  |  | **Site-pairs** | **Estimate** | **Std.error** | **p** |
| --- | --- | --- | --- | --- | --- |
| **LA - Lumen Area** | earlywood | W-D : C-D | -0.8747 | 0.5288 | 0.2546 |
|  |  | **W-D : C-M** | **-1.3464** | **0.5107** | **0.0465*** |
|  |  | C-D : C-M | -0.4717 | 0.5138 | 0.6376 |
|  | latewood | W-D : C-D | 0.2935 | 0.4996 | 0.8288 |
|  |  | W-D : C-M | -0.7730 | 0.4813 | 0.2736 |
|  |  | C-D : C-M | -1.0664 | 0.4278 | 0.0608 |
| **TB² - Conduit Reinforcement** | earlywood | W-D : C-D | 0.7646 | 0.5296 | 0.3446 |
|  |  | W-D : C-M | 0.4914 | 0.4980 | 0.5960 |
|  |  | C-D : C-M | -0.2732 | 0.4627 | 0.8272 |
|  | latewood | W-D : C-D | 0.3022 | 0.5203 | 0.8323 |
|  |  | W-D : C-M | -0.1669 | 0.4776 | 0.9352 |
|  |  | C-D : C-M | -0.4691 | 0.4429 | 0.5528 |
| **DEN - Density** | earlywood | **W-D : C-D** | **1.3626** | **0.4753** | **0.0298*** |
|  |  | W-D : C-M | 0.8377 | 0.4452 | 0.1783 |
|  |  | C-D : C-M | -0.5249 | 0.4146 | 0.4348 |
|  | latewood | W-D : C-D | -0.1065 | 0.5139 | 0.9766 |
|  |  | W-D : C-M | -0.0717 | 0.4755 | 0.9875 |
|  |  | C-D : C-M | 0.0348 | 0.3729 | 0.9952 |
| **CWT - Cell Wall Thickness** | earlywood | W-D : C-D | 0.5331 | 0.5979 | 0.6536 |
|  |  | W-D : C-M | -0.3227 | 0.5760 | 0.8429 |
|  |  | C-D : C-M | -0.8558 | 0.5410 | 0.2835 |
|  | latewood | W-D : C-D | 0.3003 | 0.5485 | 0.8493 |
|  |  | W-D : C-M | -0.2567 | 0.5146 | 0.8730 |
|  |  | C-D : C-M | -0.5570 | 0.4310 | 0.4208 |

# References

Little, E. L., and Viereck, L. A. (1971). Atlas of United States trees. U.S. Dept. of Agriculture, Forest Service.
